# Role of retinal biomarkers in diabetes detection and risk prediction: A systematic scoping review

**DOI:** 10.1016/j.dsx.2026.103405

**Published:** 2026-03-30

**Authors:** Soujanya Kaup, Sowrabha Bhat, Erin E. Reardon, Vijayaraghavan Prathiba, Ramachandran Rajalakshmi, Rodrigo M. Carrillo-Larco, Ram Jagannathan, Ranjit Mohan Anjana, Mohammed K. Ali, Viswanathan Mohan, Sudeshna Sil Kar, Anant Madabhushi, Mary Beth Weber, K.M. Venkat Narayan

**Affiliations:** a Department of Ophthalmology, Yenepoya (Deemed to be) University, Mangalore, India; b Faculty of Epidemiology and Population Health, London School of Hygiene & Tropical Medicine, Keppel Street, London, WC1E 7HT, United Kingdom; c Emory Global Diabetes Research Center, Woodruff Health Sciences Center, And Emory University, Atlanta, GA, USA; d Department of Endocrinology, Yenepoya (Deemed to be) University, Mangalore, India; e Center for Nutrition Studies, Yenepoya (Deemed to be) University, Mangalore, India; f Woodruff Health Sciences Center Library, Emory University, Atlanta, GA, USA; g Dr. Mohan’s Diabetes Specialities Centre (IDF Centre of Excellence in Diabetes Care), Chennai, India; h Dr. Rema Mohan Department of Ocular Research, Madras Diabetes Research Foundation, Chennai, India; i Madras Diabetes Research Foundation (ICMR Collaborating Centre of Excellence), Chennai, India; j Department of Family and Preventive Medicine, School of Medicine, Emory University, Atlanta, GA, USA; k Wallace H. Coulter Department of Biomedical Engineering, Emory University, Atlanta, GA, USA; l Atlanta Veterans Administration Medical Center, Atlanta, GA, USA

**Keywords:** Retinal biomarkers, Diabetes, Artificial intelligence, Systematic scoping review, Oculomics

## Abstract

**Aims:**

This systematic scoping review was conducted to map and synthesize literature on retinal biomarkers associated with type 2 diabetes (T2D), including conventional and Artificial Intelligence(AI)-derived features, while considering ethnic/geographic diversity.

**Methods:**

Following PRISMA-ScR statement, seven databases were searched up to January 28, 2026 for cross-sectional and longitudinal studies in adults (≥18 years) with or without T2D. Included studies required a normoglycemic comparator and quantitative retinal assessment, with or without AI. Two reviewers independently performed title/abstract screening, full-text review and quality appraisal (using Newcastle Ottawa scale(NOS)/modified NOS); disagreements were resolved by a third reviewer. Data extraction focused on biomarker type, methodology, and population characteristics.

**Result:**

Thirty-four studies (27 cross-sectional; 7 longitudinal) were included, mostly evaluating Caucasian/White [n = 18], Asian [n = 15] and Mixed/Latin American [n = 1] populations. Consistent vascular diabetes biomarkers included wider arteriolar and venular calibers. Arteriolar narrowing predicted incident diabetes in longitudinal analyses. Increased vessel tortuosity and altered fractal dimension were frequently observed. Ethnic variation was more pronounced in longitudinal studies; AI-based research was largely confined to China.

**Conclusions:**

Retinal biomarkers show promise for early diabetes detection, but research remains geographically skewed and requires multiethnic validation to ensure clinical impact. These findings provide the biological ground truth necessary for handcrafted feature extraction in the development of interpretative artificial intelligence.

## 1. Introduction

The number of adults living with diabetes has exceeded 800 million, yet nearly half of them remain undiagnosed [1,2]. Enhancing screening of diabetes, a significant contributor of mortality, morbidity and healthcare costs, is essential for early intervention and prevention of disease progression [3–6]. To this end, there is a growing interest in the development and implementation of non-invasive, user-friendly technologies to facilitate early detection of diabetes [7,8].

The retina has long been regarded as a ‘window’ to systemic and neurological diseases [9], offering a unique opportunity to directly and non-invasively visualize the human microvasculature [10]. Changes in the retinal vasculature have been recognized as important markers for the risk of systemic conditions, such as cardiovascular and neurological diseases [11,12]. Artificial intelligence (AI), machine learning (ML) and deep learning (DL) models applied to retinal photographs have shown potential to identify diabetes and related systemic risks, thereby expanding the role of ocular biomarkers in chronic disease diagnosis and management, an emerging field termed as oculomics [13,14]. The identification of specific retinal biomarkers not only serves in understanding the pathophysiology of diseases like diabetes, but also potentially provides a foundation for non-invasive, assistive clinical tools that facilitate opportunistic screening in primary care settings. A primary barrier to the clinical integration of AI is the ‘black-box’ nature of end-to-end deep learning, which often lacks the transparency required for medical accountability. By systematically cataloging specific retinal biomarkers, this review establishes a biological ‘ground truth’ essential for the development of inherently interpretable AI models. Unlike opaque algorithms, models built upon handcrafted, expert-driven features provide a clear diagnostic rationale, bridging the current trust gap between technology and practitioner. This shift toward interpretable oculomics is critical for fostering the clinician confidence necessary for widespread real-world adoption.

Despite the promising potential of retinal biomarkers for diabetes detection and risk stratification, there remains a notable gap in the comprehensive appraisal and synthesis of the existing evidence. To address this gap, we reviewed the literature to map existing evidence on retinal biomarkers in diabetes, including AI-based approaches and highlight opportunities for future research and clinical application. Given that this field is still evolving and highly heterogeneous, a scoping review is the most suitable approach to systematically capture, organize, and synthesize the breadth of available evidence.

This systematic scoping review is, to our knowledge, the first to systematically map specific retinal vascular and non-vascular biomarkers associated with both prevalent and incident diabetes in adults, integrating evidence from traditional quantitative image analysis and AI–based methods, while explicitly examining ethnic/geographic heterogeneity and study quality. Recent reviews instead (i) evaluate AI-models based on clinical and biochemical risk factors for predicting incident diabetes, without using retinal imaging [15], or (ii) summarize AI applied to retinal imaging to detect diabetic retinopathy or systemic diabetes-related complications (cardiovascular, kidney, neurological, etc.) rather than retinal biomarkers of diabetes itself or (iii) use retinal images to detect diabetes but without focus on specific retinal biomarkers used [16–19] Table S1 provides summary of recent reviews in this space, and highlighting the novelty of our review.

Despite the growing body of literature on oculomics, existing reviews have primarily focused on the diagnostic accuracy of specific AI models or the pathology of established diabetic retinopathy. There remains a critical gap in understanding how systemic metabolic shifts are reflected in retinal vascular and non-vascular biomarkers across diverse global populations, particularly in early type 2 diabetes (T2D). This study represents the first systematic scoping review to comprehensively map the landscape of both conventional and artificial intelligence-derived retinal biomarkers while explicitly analyzing the impact of ethnic and geographic diversity on biomarker consistency and predictive value. We hypothesize that specific retinal vascular alterations, serve as early, biomarkers of metabolic dysfunction, but that their predictive thresholds and clinical associations will exhibit significant variability across different ethnic and geographic cohorts.

The primary objectives of this systematic scoping review were to identify and map the various retinal vascular and non-vascular biomarkers associated with prevalent and incident T2D. The secondary objectives were to describe the role of artificial intelligence (AI) in biomarker extraction and to explore the impact of ethnic and geographic heterogeneity on these biomarkers.

To achieve these objectives, this review addressed the following research questions.

1. What specific retinal vascular and non-vascular structural parameters are associated with the presence of T2D?
2. Which retinal biomarkers are predictive of future T2D onset in longitudinal cohorts?
3. How is AI currently utilized for studying these biomarkers?
4. Do these retinal biomarkers vary significantly across different ethnic and geographic populations?

## 2. Methods

This systematic scoping review was reported in accordance with the Preferred Reporting Items for Systematic reviews and Meta-Analyses extension for Scoping Reviews (PRISMA-ScR) Statement. The completed reporting checklist is included as a supplementary file (Appendix 1) to ensure transparency and methodological rigor [20].

### 2.1. Review framework and registration

Following the Joanna Briggs Institute (JBI) [21] methodology for scoping reviews, the PCC (Population, Concept, Context) framework was utilized as the primary mechanism for guiding our search strategy and defining eligibility. This framework allowed us to systematically identify inclusion criteria by breaking the primary research question into three core components.

- Population: Adults (≥18 years) with or without T2D.
- Concept: Quantitative retinal vascular and non-vascular biomarkers.
- Context: All global healthcare and geographic settings to capture ethnic heterogeneity.

This review was prospectively registered in Open Science Framework (OSF) and the protocol is available on this site [registration DOI: https://doi.org/10.17605/OSF.IO/UJ5NA] [22].

### 2.2. Eligibility criteria

#### 2.2.1. Study Inclusion criteria

Guided by the PCC framework, we included original cross-sectional and longitudinal studies examining retinal vascular and non-vascular changes associated with T2D or predicting future risk of T2D among adults. Studies were included if they met the following criteria.

- Population: Included a comparator group of normoglycemic adults.
- Concept: Used direct retinal examination or fundus photography, with or without computer-assisted analysis, machine learning, or artificial intelligence (AI) tools.
- Context: Sampled community-dwelling, hospital-based, or clinic-based populations, irrespective of the ethnic/geographic location.

#### 2.2.2. Studies were excluded if they

- Focused exclusively on prediabetes, type 1 diabetes, or other specific diabetes types (gestational, drug-induced, cystic fibrosis–associated, pancreatitis-associated diabetes).
- Were published in languages other than English
- Were literature reviews
- Were clinical trials, since the review aimed to examine biomarkers reflecting natural pathological changes in T2D, not those influenced by therapeutic interventions.
- Reported combined endpoints for prediabetes and T2D where data specific to T2D could not be separately extracted or identified.

### 2.3. Information sources & search strategy

The search strategy utilized index terms and natural language keywords mapped directly to the PCC elements (e.g., combining retinal biomarkers [Concept] with T2D [Population] across all geographic settings [Context]). The search was developed (by ER) in Ovid MEDLINE and translated and run across six additional databases, Embase, Scopus, Web of Science, Cochrane Library, CINAHL, and ProQuest Dissertations & Theses, from their inception to July 17, 2024, initially. The full search strategy for all databases is available in the online supplement (Appendix 2). The searches were rerun on January 28, 2026, to capture new articles published since the original search date.

### 2.4. Source of evidence selection

The initial search results were compiled and deduplicated in EndNote 21 [23]. Retracted articles identified by EndNote’s built-in retraction detection feature were removed from the results, which were then uploaded to Covidence [24] for screening. The updated search results were also compiled in EndNote 21, retractions removed and then uploaded to Covidence for deduplication and screening. ^22^ In the first stage of screening, titles and abstracts were screened using the a priori eligibility criteria mentioned. The screening was performed independently by two reviewers (SK and SB). Articles not excluded in the title/abstract phase were moved on to the full-text screening phase. This screening was also performed by two independent reviewers (SK and SB) based on the established inclusion/exclusion criteria. Any disagreements were solved by the decision of a third reviewer (VP). To ensure the inclusion of all relevant literature, forward citation searches were conducted for the included studies. The search and screening process have been presented and reported in the form of a PRISMA flow diagram (Fig. 1).

### 2.5. Data extraction, data items, and synthesis

Following the JBI methodology [21], we developed a data extraction tool that was piloted on three articles by two reviewers (SK and SB), and additional details (such as type of fundus cameras used, details of the computer assisted tools/AI/ML/DL tools) were incorporated to improve its comprehensiveness. Data were independently extracted by two reviewers (SK and SB), with discrepancies resolved through discussion or a third reviewer (VP). In alignment with our PCC framework, data items were categorized as follows.

- Population: Study characteristics (author, year, country, design, and sample size) and T2D status/definition (American Diabetes Association/World Health Organisation diagnostic criteria) [25,26]. If diagnostic criteria for T2D were not reported, this was recorded as “not defined.” In studies including both T2D and prediabetes, only T2D-specific data were extracted.
- Concept: Retinal biomarkers (vascular or non-vascular), AI/ML/DL methods, and the type of fundus camera used.
- Context: Geographic location and ethnic characteristics of the study populations to facilitate the analysis of heterogeneity.

The analysis of the evidence in this systematic scoping review is descriptive, focusing on the role of retinal biomarkers in T2D. This approach was adopted because the available studies exhibit substantial heterogeneity in their designs, populations, outcome definitions, and analytical methodologies, making quantitative synthesis or meta-analysis infeasible. The synthesis maps the role of retinal biomarkers in T2D according to the PCC pillars, specifically summarizing findings by biomarker type (Concept), T2D status-current or future risk (Population), and ethnic/geographic variation (Context). Quantitative analysis was limited to frequencies and summary statistics.

### 2.6. Quality appraisal

The methodological quality of the included studies was appraised using the Newcastle–Ottawa Scale (NOS) for cohort (longitudinal) studies and an adapted version of the NOS for cross-sectional studies [27]. This approach evaluates quality based on star allocation across the domains of selection, comparability, and outcome, thus ensuring standardization and transparency. For cohort studies, quality was rated as ‘good,’ ‘fair,’ or ‘poor’ following the Agency for Healthcare Research and Quality (AHRQ) algorithm [28]: Good: 3 or 4 stars in selection AND 1 or 2 stars in comparability AND 2 or 3 stars in outcome; Fair: 2 stars in selection AND 1 or 2 stars in comparability AND 2 or 3 stars in outcome; and Poor: 0 or 1 star in selection OR 0 stars in comparability OR 0 or 1 star in outcome. Cross-sectional studies were appraised using the adapted NOS and classified as good (7–10 stars), satisfactory (5–6 stars), or unsatisfactory (0–4 stars) [29,30].

### 2.7. Protocol Amendments

The methodology for this scoping review was pre-specified in a protocol registered on the Open Science Framework (OSF) [22]. No significant deviations from the registered protocol occurred during the conduct of the review, except for an update to the literature search end-date. Originally slated for an earlier conclusion, the search was extended to January 28, 2026, to incorporate recent high-impact studies published in 2024 and 2025, thereby enhancing the review’s currency and comprehensiveness.

## 3. Results

The search yielded 26961 (25751from the databases and an additional 1210 from citation searches). After removing duplicates, 12259 potentially relevant studies remained for title/abstract screening. The title and abstract screening resulted in 851 full-text articles. Of these, we selected 34 original articles that fulfilled the eligibility criteria (Fig. 1). Following PRISMA-ScR transparency standards (Item 17) (Appendix 1), the 816 exclusions were categorized narratively to clarify the review’s scope: Population/Comparator Mismatch: Studies were excluded if they lacked a normoglycemic control group or focused exclusively on Type 1 Diabetes or other specific types (e.g., gestational). Methodological/Concept Gaps: Exclusions included literature reviews, clinical trials (as the focus was on natural pathological biomarkers rather than therapeutic interventions), and studies where T2D data could not be isolated from prediabetes. Publication Type: Non-English publications were excluded to ensure consistent data extraction and synthesis.

### 3.1. Characteristics of included studies

Following the PCC framework, the 34 included studies, published between 2002 and January 2026, were synthesized according to their primary focus:

Population: The review analyzed evidence from 27 cross-sectional (79.4%) [31–57] (investigated associations of retinal biomarkers with the current diagnosis of T2D); and 7 longitudinal (20.6%) [58–64] studies (identified baseline retinal biomarkers predicting future development of T2D among normoglycemic individuals). Eleven of the 34 studies included participants with prediabetes along with T2D or assessed prediabetes; however, only data relevant to T2D were used for this review. [36,41,44,46,49,56,58–60,65,66]. The main characteristics of the included publications are described in Table S2.

Concept: Synthesis focused on quantitative retinal vascular (n = 31) and non-vascular biomarkers (n = 3), primarily extracted via computer-assisted (n = 27) or AI/ML-based (n = 7) methodologies.

Context: The studies originated (Fig. 2) mainly from high-income countries (n = 23 [67.6%], Australia [8] [37,44–46,49,59,60,64], Greece [1] [42,67], Netherlands [3] [39,51,58], Norway [1] [48], Poland [1] [40], Singapore [5] [31,34,35,47,61], United Kingdom [1] [43], United States [3]) [41,62,63]. Upper Mid income countries contributed towards 23.5% of the publications (n = 8-China [7] [33,36, 38,50,52,53,57] and Brazil [1]) [32]. There were three publications from a Low-mid income country (8.2%- India [3]) [54–56].

### 3.2. Methodological quality of the evidence base

“While a scoping review typically provides a descriptive synthesis without weighting results, we utilized the **Newcastle–Ottawa Scale (NOS)** to characterize the methodological rigor of the evidence. This appraisal reveals a high-quality foundation for longitudinal research, with 100% (n = 7/7) of such studies judged as ‘Good’. Conversely, 33.3% (n = 9/27) of cross-sectional studies scored lower, primarily due to limited sample representativeness or a lack of sample size justification (Supplementary Table S3). By reporting these quality metrics alongside our findings, we provide a transparent overview of the review, satisfying the PRISMA-ScR requirement for transparent reporting of evidence quality.

### 3.3. Retinal vascular features in T2D

The included studies comprehensively evaluated several retinal vascular biomarkers measurable on fundus photography, providing a robust overview of structural microvascular alterations associated with T2D across diverse adult populations regardless of ethnicity or geographic location. Table 1 summarizes the major retinal biomarkers studied.

Vessel caliber changes (arteriolar and venular diameters) were measured in most of the studies (n = 29/34; 85%). Some studies explicitly stated the methods used to measure the mean vessel caliber such as Central Retinal Arteriolar Equivalent (CRAE) and Central Retinal Venular Equivalent (CRVE) [31]. Most studies reported wider arteriolar caliber (n = 13/29; 45%) followed by wider venular caliber (n = 9/29; 31%). Arteriolar narrowing was reported by a minority of the studies (n = 6/29; 21%). None of the studies reported narrowing of venules.

Retinal vessel caliber in T2D (Cross-sectional Findings): Findings from cross-sectional studies reflected a pattern similar to the overall literature, with arteriolar and venular widening being the most consistent vascular features associated with the current diagnosis of T2D.

Retinal vessel caliber for predicting future risk of T2D (Longitudinal Findings): In contrast, studies designed to predict future risk of T2D demonstrated a distinct biomarker profile. Arteriolar narrowing was more consistently associated with the development of incident T2D, emerging as the predominant predictor in 43% (3 out of 7) of longitudinal studies [24,30,31].

The Arteriolar-to-Venular Ratio (AVR) summarizes relative changes in vessel calibers by dividing CRAE by CRVE. Among seven studies that measured AVR, two (28.6%) reported significant associations with T2D ([33,68]) (one longitudinal study and one cross-sectional study), showing lower AVR in participants with T2D, while five studies found no significant association.

Vessel tortuosity quantifies the degree of vessel curvature, calculated as the ratio between the actual vessel length and the straight-line distance between endpoints [54]. Increased tortuosity represents abnormal vascular remodeling caused by diabetes-induced microvascular damage. Seven out of ten cross-sectional studies (70%) reported significantly increased vessel tortuosity in T2D [31,43,45,50,51,54,56,57], although statistical significance sometimes diminished after adjusting for confounders [43]. One longitudinal study evaluated vessel tortuosity as a predictive biomarker for incident T2D but did not identify a significant association [61].

The fractal dimension measures the spatial complexity and branching density of the retinal vascular network [38,55]. A decreased fractal dimension indicates vascular rarefaction and impaired microvascular architecture. Five of ten cross-sectional studies reported significant alterations in fractal dimension among participants with T2D, although findings showed mixed directions [36,49,50,55,57]. One longitudinal study evaluated the role of fractal dimensions in prediction of T2D but found no significant associations [61].

Other Vascular Parameters: Less frequently studied markers, including vascular branching angles, vessel area, length density, and fractal branching coefficients, also showed significant structural alterations across fewer studies, indicating a widespread impact of T2D on retinal microvascular morphology.

### 3.4. Non-vascular retinal biomarkers in T2D

The non-vascular biomarker domain centered on retinal oxygen metabolism metrics including oxygen saturation [33] and optical density ratios (ODR) [52,53], measured using oximetry software and advanced AI-based image analysis such as convolutional neural networks. Elevated retinal vessel oxygenation (detected directly by oximetry or indirectly through higher ODR)- was consistently observed in T2D, including in subjects without clinically detectable retinopathy, offering complementary insights into early retinal metabolic impairment.

### 3.5. Retinal biomarkers identified using artificial intelligence (AI) associated with T2D

Most included studies [40,58,61,69] used established computer-assisted or traditional image analysis methods (such as IVAN, VISUALIS, VesselMap, SIVA, or RHINO software) to automatically or semi-automatically quantify retinal vascular parameters, using rule-based algorithms or manual verification rather than AI or ML. The following section focuses on studies that specifically leveraged AI/ML/DL-based analysis, as distinct from the more widely adopted computer-assisted, algorithmic, or semi-automated image processing methods.

Only seven studies were identified that applied AI/ML/DL techniques to quantify retinal biomarkers associated with T2D (Table 2). Fig. S1 shows an example of a retinal colour photograph and the use of artificial intelligence (AI)/machine learning (ML) tools for vessel segmentation, artery–vein classification, and feature extraction. AI was used in two main ways: (i) AI (DL)-assisted segmentation and feature extraction, where vascular or non-vascular parameters were automatically quantified, and (ii) end-to-end AI (ML and DL) classification pipelines, which directly predicted T2D status.

- AI-assisted (DL–based) segmentation and feature extraction: Several studies used DL–based segmentation approaches such as Dense-block GANs [38], U-Nets with Inception-V3 backbones [36], U-Net with PyTorch pipelines [53], HR-Net (High--Resolution Network) [57], Patch-based U-Net with nnU-Net and Linear Discriminant Analysis (LDA) [56] to extract vascular parameters including vessel diameters, fractal dimensions, tortuosity, branching angles, vessel density, and vessel area ratios. Two additional studies [52,53] applied U-Net–based models to quantify the optical density ratio (ODR), a surrogate for retinal oxygen saturation, demonstrating its potential as a non-vascular biomarker of T2D.
- AI-based (machine learning and deep learning–based) classification pipelines: One study by Zee et al., [50] developed a comprehensive ML framework that combined a convolutional neural network (CNN) (InceptionResNet) with support vector machines (SVMs). The CNN automatically extracted a range of retinal features (including vessel geometry, fractal, texture, and spectral characteristics) which was then used by the SVM to predict T2D status. Unlike studies that applied AI solely for segmentation and biomarker quantification, this approach integrated feature extraction and classification into a single predictive framework.

### 3.6. Context - ethnic differences in retinal biomarkers associated with T2D

Most studies were among predominantly Caucasian/White populations (n = 18; 53%), followed by Asians (n = 15; 44%), with relatively few studies from other major global ethnic groups, highlighting an important gap in the literature.

As illustrated in Fig. 3, the most consistent retinal biomarkers associated with prevalent T2D in both Caucasian and Asian populations, based on cross-sectional analyses, were wider arteriolar and venular caliber. Notable ethnic differences emerged in longitudinal research examining predictors of incident T2D: narrowed arteriolar caliber were the most frequently reported predictor in Caucasian populations (3 out of 6 studies; 50%) [60,62,63], while the sole longitudinal study in Asian participants identified wider arteriolar and venular calibers as predictive markers of T2D onset [61].

Studies examining arteriole-to-venule ratio (AVR) indicate ethnic variability. Findings among Asian populations were mixed, with some studies reporting a smaller AVR in T2D and others finding no significant difference. In contrast, most Caucasian cohorts (including studies from Greece, the UK, and the Netherlands) did not demonstrate significant differences in AVR between T2D and non-T2D groups [43,51,67]. Notably, a longitudinal study from the United States found that a lower AVR at baseline predicted incident T2D, highlighting its potential prognostic value in that setting [63].

Cross-sectional studies conducted in Caucasian populations consistently demonstrated an association between increased retinal vessel tortuosity and T2D [45,51], though in some cases this association lost statistical significance after adjustment for confounders [43]. Among studies in Asians, findings were more variable, with some reporting increased tortuosity in T2D(31,50,51,54) and others not observing a significant association. Overall, the relationship between vessel tortuosity and T2D appears more robust in cross-sectional studies of Caucasian cohorts compared to Asian populations, where greater inconsistency is observed.

A synthesis of available studies shows that, at the population level, Chinese cohorts consistently demonstrate reduced fractal dimensions and diminished retinal branching geometry in T2D, reflecting decreased microvascular complexity [36,38,50]. In contrast, studies from India and Singapore, as well as from Australia and the UK, present more mixed findings: some noting increased fractal dimension or branching parameters [49,55], others no significant differences [31,43]. Singaporean studies including multi-ethnic samples (Chinese, Malays, Indians) that studied the future prediction of T2D, also tended toward no significant association [61]. Overall, Chinese studies display the most uniform trend with reduced fractal dimensions, with other populations showing less consistency.

In multi-ethnic U.S. cohorts, ethnic-specific patterns were evident. Wider arteriolar caliber in T2D was observed predominantly among Whites [41,64], while wider venular caliber was associated with T2D in Hispanic and Chinese participants. These associations were weak or absent among African Americans [41].

## 4. Discussion

This systematic scoping review highlights the identification of specific retinal biomarkers that may be associated with T2D, measured using widely accessible fundus cameras. Retinal vascular biomarkers provide diagnostic value and may identify persons at risk of future development of T2D, although their expression appears to differ by ethnicity. Most research originates from high- and upper-middle-income countries, with AI-based retinal biomarker studies concentrated primarily in China, revealing important geographic disparities. This underscores the urgent need to validate biomarkers and AI models in diverse, multiethnic populations and expand research in underrepresented regions.

Our review highlights several consistent retinal vascular alterations associated with T2D. Notably, wider arteriolar and venular diameter was frequently observed, whereas arteriolar narrowing predicted incident T2D in multiple longitudinal studies, more commonly among Caucasian populations. Increased AVR was not a consistent finding and provides limited standalone discriminative value in T2D. Increased vessel tortuosity was a common finding across ethnicities. Parameters such as fractal dimension and vascular branching showed variable patterns but generally reflected disrupted retinal microvascular architecture in T2D. Non-vascular biomarkers related to retinal oxygen saturation were infrequently studied, likely due to our focus on fundus camera imaging.

Importantly, there was some variations in retinal biomarker findings across ethnicities predominantly in longitudinal studies predicting future T2D, while cross-sectional studies showed broadly similar patterns of arteriolar and venular dilation regardless of ethnicity. These differences may reflect true ethnic variability but could also be influenced by methodological heterogeneity in study design, imaging protocols, and measurement techniques.

The observed pattern of arteriolar narrowing prior to the onset of T2D, followed by both arteriolar and venular dilation after T2D develops, reflects distinct underlying pathophysiological mechanisms at different disease stages. In the early stages of T2D, endothelial dysfunction is characterized by an imbalance between vasoconstrictors such as endothelin-1 and vasodilators like nitric oxide, contributes to vascular stenosis and arteriolar narrowing in the retina [70]. Hyperglycemia-induced oxidative stress and activation of pathways such as polyol and Protein Kinase-C exacerbate these effects [70]. Progressive loss of pericytes and vascular smooth muscle cells impairs vascular autoregulation, and as T2D advances, venular dilation becomes more prominent, reflecting cumulative vascular dysfunction [70].

AI-assisted retinal biomarker research for T2D is largely limited to Chinese populations, revealing geographic gaps in global studies. This predominance may reflect better strategies for organizing, sharing, and integrating large-scale retinal imaging datasets in China, which facilitate the development and validation of robust AI models compared to other regions. Most existing AI work has focused on segmentation and feature quantification rather than developing integrated classification pipelines, underscoring a critical gap. Although end-to-end DL models have demonstrated impressive T2D classification performance (AUC range: 0.80–0.92) [71–74], these models often act as ‘black boxes,’ offering limited insight into the specific retinal features driving predictions. The use of attention maps or gradient-based saliency techniques suggest that these models frequently rely on vascular (and occasionally non-vascular) features. However, as these studies did not explicitly report or validate the specific retinal biomarkers used, they were not included in the biomarker-based synthesis of this review. We acknowledge, however, that this approach may exclude some studies whose algorithms likely use vasculature and highlight the broader interpretability and transparency challenges in AI-based retinal research. In contrast, machine learning using expert-defined retinal features achieves strong accuracy with smaller datasets, with better interpretability, and reduces reliance on large, annotated datasets, making it ideal for resource-limited settings [75–78].

### 4.1. Perspectives for clinical and assistive practice

The use of AI on retinal images has a potential to revolutionize not only detection of T2D complications such as diabetic retinopathy, neuropathy, nephropathy, and atherosclerotic cardiovascular disease, as well as to predict the risk of cardiovascular events but also in detection of current and future risk of T2D [18]. The rapid proliferation of DL in oculomics has introduced a significant “trust gap” in clinical practice. Most contemporary DL models operate as “black boxes,” where end-to-end pixel analysis yields a diagnostic output without an auditable physiological rationale. To address this, many researchers utilize post-hoc interpretability tools, such as Saliency Maps or Gradient-weighted Class Activation Mapping (Grad-CAM). However, these “heat maps” are frequently criticized for lacking scientific validity [79]. They often identify image artifacts or non-specific regions as “areas of interest,” providing a visual justification that may not correspond to actual pathophysiology. To bridge this gap, there is a burgeoning shift toward inherently interpretable AI models. Unlike “black boxes,” these systems are built upon expert-driven, handcrafted features, such as vessel tortuosity, branching coefficients, AVR etc. When an AI model is trained on these specific, biologically grounded parameters, it provides an auditable diagnostic rationale. By systematically cataloging vascular and non-vascular biomarkers associated with both prevalent and incident T2D, we provide the biological ground truth necessary for handcrafted feature extraction for development of interpretative AI. By standardizing these biomarkers across diverse ethnic and geographic populations, this review provides the blueprint for assistive clinical tools that can be integrated into primary care. Such inherently interpretable systems ensure that AI serves to assist clinical judgment, but as a transparent, verifiable partner in closing the global diagnostic gap for T2D.

### 4.2. Strengths and limitations

A key strength of this review is the rigorous systematic methodology employed, following established guidelines such as PRISMA-ScR statement and the Joanna Briggs Institute (JBI) methodology guidance for scoping reviews [20,21], with searches spanning multiple databases. The systematic extraction and synthesis process enhances the review’s comprehensiveness, reproducibility, and transparency, providing a robust foundation for identifying research gaps and guiding future studies in this evolving field.

Limitations include a restriction to English-language publications, which may potentially omit relevant studies. Crucially, as there was a paucity of data from major global regions (Africa, South America, and parts of Central Asia etc), the findings may lack universal generalizability and primarily reflect the populations currently overrepresented in oculomics research. Additionally, heterogeneity in study design and imaging protocols posed challenges for quantitative synthesis, leading to the rationale for adopting a scoping review approach to comprehensively map the breadth of evidence.

### 4.3. Conclusions and recommendation for future research

In conclusion, understanding retinal biomarkers not only deepens insight into the microvascular pathophysiology of T2D but establishes the retina as a vital ‘window’ for early detection through AI-driven oculomics. This scoping review systematically maps the current evidence base, highlighting the significant promise of specific vascular features while uncovering critical geographic and ethnic disparities. Future research must prioritize the transition from ‘black-box’ algorithms to inherently interpretable AI frameworks that utilize the expert-driven biomarkers such as those cataloged here. Such transparency is essential for building clinician trust and ensuring that automated diagnoses are biologically grounded. Furthermore, the integration of cloud-based imaging systems and emerging technologies, like Generative AI and Large Vision Models, offers a scalable frontier for predictive oculomics. Finally, prospective, multi-ethnic validation is required to move toward a truly global, equitable, and non-invasive approach to T2D screening.

## 5. Twitter summary

Can our eyes predict diabetes before it starts? Our new review maps retinal biomarkers that signal early risk. Identifying these specific features helps move beyond “black-box” AI toward transparent, interpretable tools for more trustworthy global screening.

## Supplementary Material

1

## Figures and Tables

**Fig. 1.**
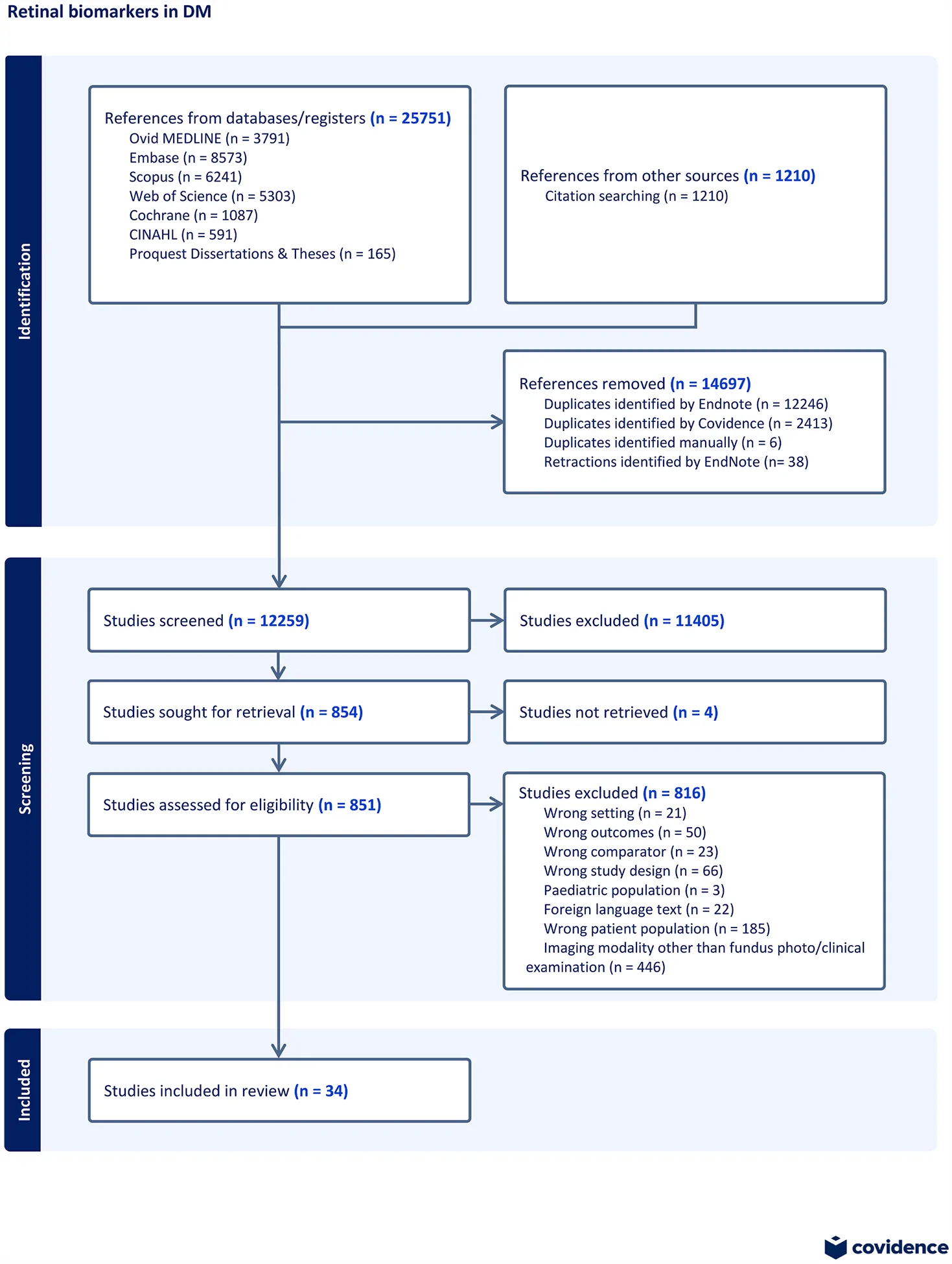
Flow diagram of literature search and selection based on Preferred Reporting Items for Systematic Reviews and Meta-analyses (PRISMA).

**Fig. 2.**
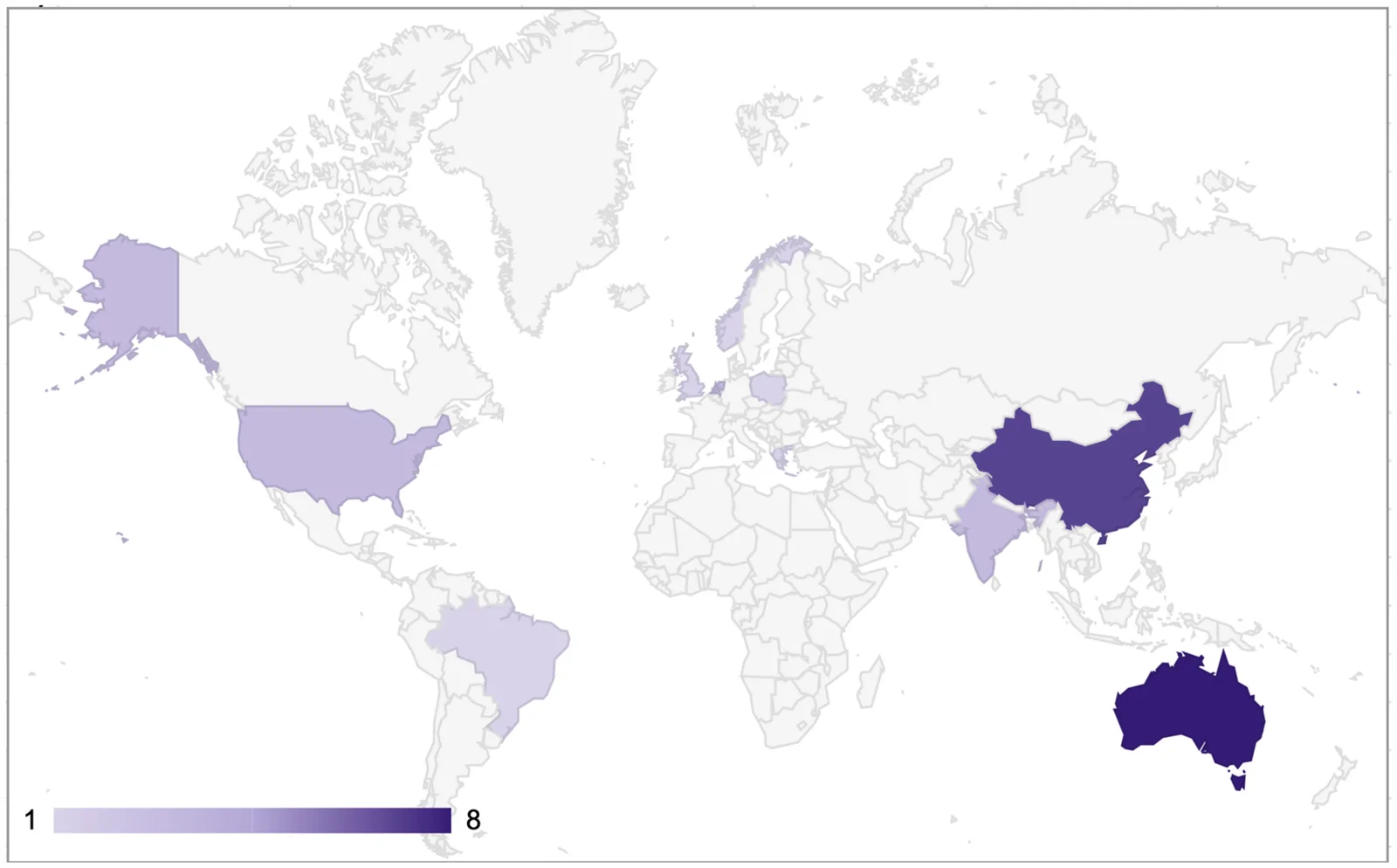
Geographic distribution of publications on retinal biomarkers in type 2 diabetes, shown by country with intensity scale (1–8 publications).

**Fig. 3.**
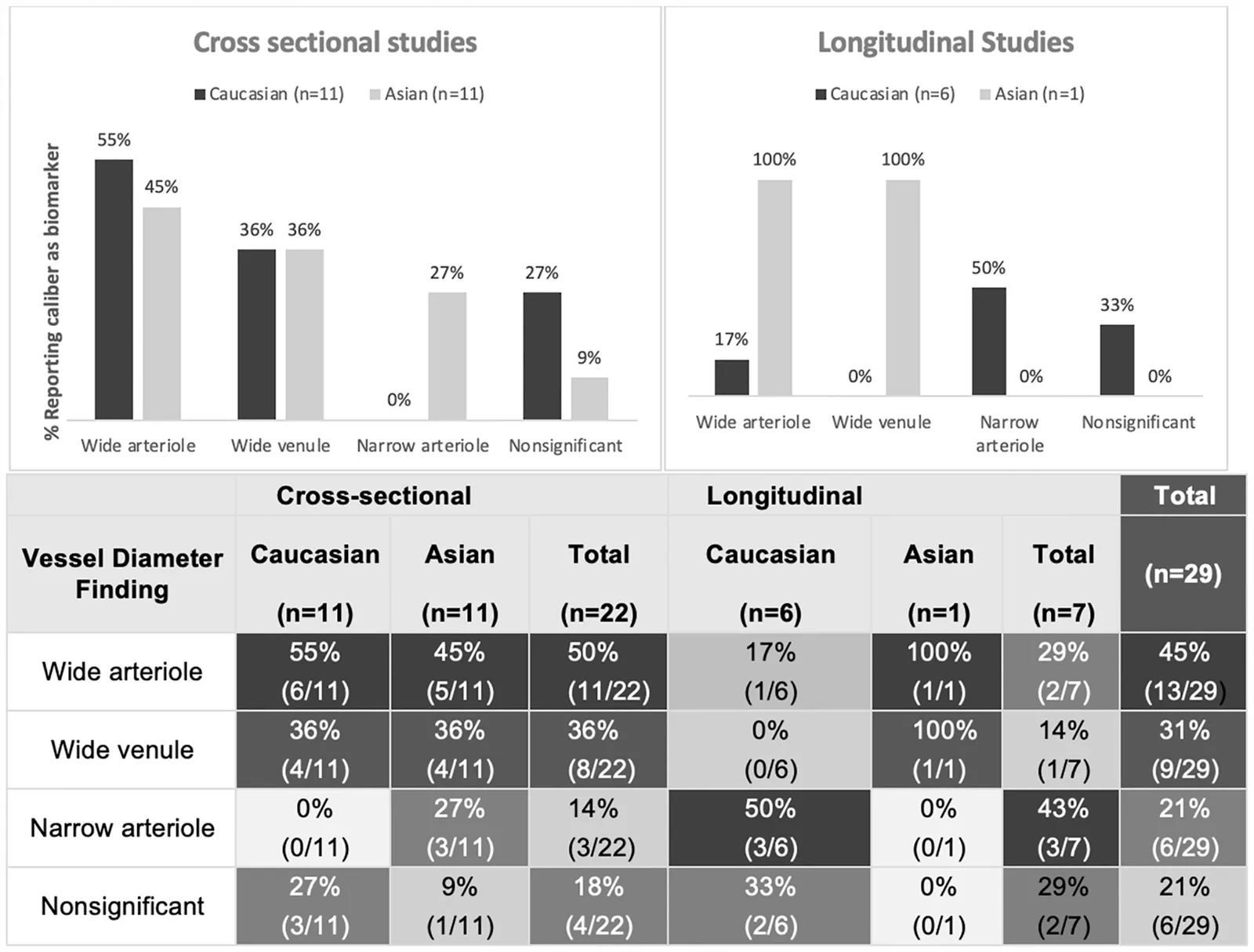
Bar graphs indicate the proportion of cross-sectional and longitudinal studies reporting key retinal vessel findings among Caucasian and Asian populations. Table summarizes corresponding study proportions and their findings. [Footnote: Percentages represent the proportion of studies that identified each retinal vessel biomarker as statistically significant. The numbers in parentheses indicate the number of studies reporting a positive association (numerator) out of the total number of studies that evaluated the particular vessel diameter finding (denominator)*Five studies did not study average vessel caliber *The combined percentage total exceeds 100% because several studies reported more than one statistically significant vessel caliber finding (e.g., both wider arterioles and wider venules). These overlapping results reflect studies that contributed findings to multiple categories, as shown in the table above. There were no representative studies on vessel caliber from other ethnicities.].

**Table 1:** Summary of vascular and non-vascular retinal biomarkers associated with or predictive of type 2 diabetes in included studies (n = 34) from the systematic review.

Biomarker	Definition	Cross-Sectional (n, %)	Longitudinal (n, %)	Diagnostic/Predictive Relevance
Arteriolar Caliber (CRAE)	Central Retinal Arteriolar Equivalent; mean arteriole diameter	11/22 (50%) wider in DM; 3/22 (14%) narrower	2/7 (29%) wide arteriolar and 3/7 (43%) arteriolar narrowing predictive	Arteriolar caliber (CRAE) is the most consistently reported retinal biomarker associated with T2D. Approximately half of cross-sectional studies identified significantly wider arteriolar caliber in individuals with T2D, while nearly half of longitudinal studies found that narrower arterioles independently predicted the development of T2D.
Venular Caliber (CRVE)	Central Retinal Venular Equivalent; mean venule diameter	8/22 (36%) wider in DM	1/7 (14%) predictive value (Asian study)	Venular caliber was the second most frequently reported retinal biomarker to show a significant association with T2D, with wider venules documented in more than one-third of studies. However, its prognostic value is limited, as only a single longitudinal study found wider venular caliber to be predictive of incident T2D.
AVR (CRAE/CRVE ratio)	Ratio of CRAE to CRVE	1/5 (20%) lower in DM	One longitudinal study showed lower AVR predictive of DM	AVR was assessed in six studies but showed inconsistent and mostly non-significant associations with T2D, limiting its value as a retinal biomarker for detection or risk prediction.
Vessel Tortuosity	Ratio of vessel path length to straight distance	Increased vessel tortuosity in 7/10 (70%) of studies, though one study lost statistical significance after adjusting for confounders.	Assessed in only one study, but was not predictive of DM.	Vessel tortuosity was increased in most studies of T2D; no longitudinal studies assessed its predictive value. It serves as a marker of abnormal retinal vascular remodeling.
Fractal Dimension	Complexity/branching measure of vessel network	5/10 (56%) significant change (lower in 4/10 (40%) and higher in 2/10 (20%))	Assessed in only one study, but was not predictive of DM.	Fractal dimension showed significant but mixed directional changes, with lower values in 40% and higher values in about one-fifth of studies, reflecting heterogeneity in T2D-associated vascular remodeling.
Retinal Oxygenation/ODR (Optical Density Ratio)	Measures oxygen saturation within vessels	2/2 (100%) elevated in DM	Not Assessed	Reflects early metabolic dysfunction

Please note: The denominators for each biomarker vary, as not all biomarkers were evaluated in every included study. T2D = Type 2 Diabetes.

**Table 2:** Use of Artificial Intelligence (AI) methods among studies included in the systematic review of retinal biomarkers in type 2 diabetes.

Study (Year)	AI Method	AI Role Category
Li et al (2022, China) [38]	Dense-block GAN (D-GAN) + custom software	AI-assisted segmentation/feature extraction
Jiang et al (2024, China) [36]	*U-Net* with Inception-V3 backbone	AI-assisted segmentation/feature extraction
Zee et al (2022, China) [50]	*ARIA* program + *InceptionResNet* CNN + SVM	Full AI/ML classification pipeline
Zhang et al (2023, China) [52]	*U-Net* + multipath attention network (MA-net)	AI-assisted segmentation/feature extraction
Zhang et al (2024, China) [53]	*U-Net + PyTorch*	AI-assisted segmentation/feature extraction
Chen et al (2025, China) [57]	*HR-Net* (High-Resolution Network)	AI-assisted segmentation/feature extraction
Kaup et al (2026, India) [56]	Patch-based *U-Net* + *nnU-Net* + Linear Discriminant Analysis (LDA)	AI-assisted segmentation/feature extraction

GAN: Generative Adversarial Network; U-Net: Convolutional neural network architecture for image segmentation; CNN: Convolutional Neural Network; SVM: Support Vector Machine; AI: Artificial Intelligence; ML: Machine learning.

## What is known about this research topic?

Type 2 diabetes is a major global health burden often diagnosed only after the onset of irreversible microvascular and macrovascular complications.

The retinal vasculature provides a unique, non-invasive window into systemic health, reflecting early metabolic changes before clinical symptoms appear.

## What this study adds and its future implications?

This review maps consistent biomarkers like wider vessel calibers while revealing major geographic gaps and ethnic variability, such as arteriolar narrowing predicting new-onset disease specifically in Caucasian populations.

We identify that most artificial intelligence research remains limited to vessel segmentation, with a critical lack of end-to-end pipelines for direct clinical diagnosis.

The identification of these specific biomarkers enables the development of interpretable artificial intelligence tools that are inherently transparent and avoid “black-box” limitations.

These findings support the integration of automated, ethnic-specific retinal screening into primary care as a trustworthy, assistive tool for early metabolic risk stratification.

## Appendix A. Supplementary data

Supplementary data to this article can be found online at https://doi.org/10.1016/j.dsx.2026.103405.
