# Supplementary material for: Role of retinal biomarkers in diabetes detection and risk prediction: A systematic scoping review
Source: Diabetes Metab Syndr. Author manuscript; Available in PMC 2026 Nov 16. (PMC13770757; doi:10.1016/j.dsx.2026.103405)
Supplement: Supplementary file 1 [file NIHMS2221938-supplement-1.docx]

# Table of contents

| Sl. No | Content | Page number |
| --- | --- | --- |
| 1. | Appendix 1: Preferred Reporting Items for Systematic reviews and Meta-Analyses extension for Scoping Reviews (PRISMA-ScR) Checklist | 2-3 |
| 2. | Appendix 2: Search Strategy | 4-9 |
| 3. | Table S1: Table summarizing related systematic and scoping reviews | 8-9 |
| 4. | Table S2: Overview of Cross-Sectional and Longitudinal Studies Examining Retinal Biomarkers in type 2 diabetes | 10-26 |
| 5. | Table S3: Critical Appraisal of included Cross-sectional Studies Using the Modified Newcastle–Ottawa Scale | 27-39 |
| 6. | Figure S1. Example of a retinal colour photograph and the use of artificial intelligence (AI)/machine learning (ML) tools for vessel segmentation, artery–vein classification, and feature extraction | 30 |

**Appendix 1: Preferred Reporting Items for Systematic reviews and Meta-Analyses extension for Scoping Reviews (PRISMA-ScR) Checklist**

| **SECTION** | **ITEM** | **PRISMA-ScR CHECKLIST ITEM** | **REPORTED ON PAGE #** |
| --- | --- | --- | --- |
| **TITLE** | | | |
| Title | 1 | Identify the report as a scoping review. | 1 |
| **ABSTRACT** | | | |
| Structured summary | 2 | Provide a structured summary that includes (as applicable): background, objectives, eligibility criteria, sources of evidence, charting methods, results, and conclusions that relate to the review questions and objectives. | 3 |
| **INTRODUCTION** | | | |
| Rationale | 3 | Describe the rationale for the review in the context of what is already known. Explain why the review questions/objectives lend themselves to a scoping review approach. | 6 |
| Objectives | 4 | Provide an explicit statement of the questions and objectives being addressed with reference to their key elements (e.g., population or participants, concepts, and context) or other relevant key elements used to conceptualize the review questions and/or objectives. | 6-7 |
| **METHODS** | | | |
| Protocol and registration | 5 | Indicate whether a review protocol exists; state if and where it can be accessed (e.g., a Web address); and if available, provide registration information, including the registration number. | 7 |
| Eligibility criteria | 6 | Specify characteristics of the sources of evidence used as eligibility criteria (e.g., years considered, language, and publication status), and provide a rationale. | 7-8 |
| Information sources* | 7 | Describe all information sources in the search (e.g., databases with dates of coverage and contact with authors to identify additional sources), as well as the date the most recent search was executed. | 8 |
| Search | 8 | Present the full electronic search strategy for at least 1 database, including any limits used, such that it could be repeated. | 8 and appendix 1 of online supplement |
| Selection of sources of evidence† | 9 | State the process for selecting sources of evidence (i.e., screening and eligibility) included in the scoping review. | 8 |
| Data charting process‡ | 10 | Describe the methods of charting data from the included sources of evidence (e.g., calibrated forms or forms that have been tested by the team before their use, and whether data charting was done independently or in duplicate) and any processes for obtaining and confirming data from investigators. | 9-10 |
| Data items | 11 | List and define all variables for which data were sought and any assumptions and simplifications made. | 9-10 |
| Critical appraisal of individual sources of evidence§ | 12 | If done, provide a rationale for conducting a critical appraisal of included sources of evidence; describe the methods used and how this information was used in any data synthesis (if appropriate). | 10 and Table S3 (online supplement) |
| Synthesis of results | 13 | Describe the methods of handling and summarizing the data that were charted. | 7 |
| **RESULTS** | | | |
| Selection of sources of evidence | 14 | Give numbers of sources of evidence screened, assessed for eligibility, and included in the review, with reasons for exclusions at each stage, ideally using a flow diagram. | Figure 1 |
| Characteristics of sources of evidence | 15 | For each source of evidence, present characteristics for which data were charted and provide the citations. | 11 |
| Critical appraisal within sources of evidence | 16 | If done, present data on critical appraisal of included sources of evidence (see item 12). | 12 and Table S3 (online supplement) |
| Results of individual sources of evidence | 17 | For each included source of evidence, present the relevant data that were charted that relate to the review questions and objectives. | Table S2 (online supplement) |
| Synthesis of results | 18 | Summarize and/or present the charting results as they relate to the review questions and objectives. | 12-17 |
| **DISCUSSION** | | | |
| Summary of evidence | 19 | Summarize the main results (including an overview of concepts, themes, and types of evidence available), link to the review questions and objectives, and consider the relevance to key groups. | 17-18 |
| Limitations | 20 | Discuss the limitations of the scoping review process. | 20 |
| Conclusions | 21 | Provide a general interpretation of the results with respect to the review questions and objectives, as well as potential implications and/or next steps. | 21 |
| **FUNDING** | | | |
| Funding | 22 | Describe sources of funding for the included sources of evidence, as well as sources of funding for the scoping review. Describe the role of the funders of the scoping review. | 21 |

*From:* Tricco AC, Lillie E, Zarin W, O'Brien KK, Colquhoun H, Levac D, et al. PRISMA Extension for Scoping Reviews (PRISMAScR): Checklist and Explanation. Ann Intern Med. 2018;169:467–473. [doi: 10.7326/M18-0850](http://annals.org/aim/fullarticle/2700389/prisma-extension-scoping-reviews-prisma-scr-checklist-explanation).

Appendix 2: Search Strategy

### Databases:

1. Ovid Medline - 3351
2. Embase - 7210
3. Scopus - 5417
4. Web of Science - 4745
5. Cochrane - 1001
6. CINAHL - 564
7. ProQuest Dissertations & Theses Global - 165

**Searches executed:** 7/17/2024

**Total search results:** 22453

Total duplicates removed in Endnote: 12246

Retractions removed in Endnote: 31

### Updated Search:

Totals listed below are additional records added to these databases since July 17, 2024.

1. Ovid Medline - 440
2. Embase - 1363
3. Scopus - 824
4. Web of Science - 558
5. Cochrane - 86
6. CINAHL - 27
7. ProQuest Dissertations & Theses Global - 0

**Searches executed:** 1/28/2026

Retractions removed in Endnote 2025: 7

**Total search results uploaded to Covidence:** 3298

### Ovid Medline (3351)

| **#** | **Search Statement** | **Results** |
| --- | --- | --- |
| 1 | ((retina* adj2 ((capillar* adj2 (dense or density)) or ((arterio-venous or artery or arterial or arteriolar or arteriole* or vein or venous or venular or venule* or vasculature or vascular or microvascular or macrovascular or vessel*) adj2 (change* or alteration* or ratio* or diameter* or narrow* or dilat* or caliber or calibre or geometry or geometrical or tortuosity or tortuous or fractal dimension or branching angle or parameter* or architecture*)))) or (macula* adj2 (thin* or thick*)) or foveal avascular zone or peripapillary retinal nerve fiber* or parafoveal capillary).ti,ab,kw,kf. | 13019 |
| 2 | exp Diabetes Mellitus/ or exp Prediabetic State/ | 530369 |
| 3 | (diabet* or prediabet* or pre-diabet* or (glucose adj1 (tolerance or intolerance))).ti,ab,kw,kf. | 854550 |
| 4 | 2 or 3 | 914076 |
| 5 | 1 and 4 | 3274 |
| 6 | (retina* adj3 (marker* or biomarker or alteration* or damage* or change* or complicat* or abnormal* or function* or dysfunction*)).ti,ab,kw,kf. | 31657 |
| 7 | ((diabetes or prediabetes or prediabetic or pre-diabetes or pre-diabetic or (glucose adj1 (tolerance or intolerance))) adj5 (risk or risks or incidence* or predict* or associat* or correlate*)).ti,ab,kw,kf. | 137659 |
| 8 | 6 and 7 | 404 |
| 9 | 5 or 8 | 3574 |
| 10 | exp adult/ or exp adolescent/ or exp child/ or exp infant/ | 10339330 |
| 11 | exp adult/ | 8128846 |
| 12 | 10 not 11 | 2210484 |
| 13 | exp humans/ or exp animals/ | 27344115 |
| 14 | exp humans/ | 22103416 |
| 15 | 13 not 14 | 5240699 |
| 16 | 12 or 15 | 7450825 |
| 17 | 9 not 16 | 3351 |

### Embase (7210)

((retina* AND ((capillar* NEAR/2 (dense OR density)) OR (('arterio venous' OR artery OR arterial OR arteriolar OR arteriole* OR vein OR venous OR venular OR venule* OR vasculature OR vascular OR microvascular OR macrovascular OR vessel*) NEAR/2 (change* OR alteration* OR ratio* OR diameter* OR narrow* OR dilat* OR caliber OR calibre OR geometry OR geometrical OR tortuosity OR tortuous OR 'fractal dimension' OR 'branching angle' OR parameter* OR architecture*))) OR (macula* NEAR/2 (thin* OR thick*)) OR 'foveal avascular zone' OR 'peripapillary retinal nerve fiber*' OR 'parafoveal capillary') AND ('diabetes mellitus'/exp OR 'impaired glucose tolerance'/exp OR diabet* OR prediabet* OR 'pre diabet*' OR (glucose NEAR/1 (tolerance OR intolerance))) OR ((retina* NEAR/3 (marker* OR biomarker OR alteration* OR damage* OR change* OR complicat* OR abnormal* OR function* OR dysfunction*)) AND ((diabetes OR prediabetes OR prediabetic OR 'pre diabetes' OR 'pre diabetic' OR 'glucose tolerance' OR 'glucose intolerance') NEAR/5 (risk OR risks OR incidence* OR predict* OR associat* OR correlate*)))) NOT (('adult'/exp OR 'juvenile'/exp OR 'infant'/exp) NOT 'adult'/exp OR (('human'/exp OR 'animal'/exp) NOT 'human'/exp))

### Scopus (5417)

( ( TITLE-ABS-KEY ( ( retina* W/2 ( ( capillar* W/2 ( dense OR density ) ) OR ( ( arterio-venous OR artery OR arterial OR arteriolar OR arteriole* OR vein OR venous OR venular OR venule* OR vasculature OR vascular OR microvascular OR macrovascular OR vessel* ) W/2 ( change* OR alteration* OR ratio* OR diameter* OR narrow* OR dilat* OR caliber OR calibre OR geometry OR geometrical OR tortuosity OR tortuous OR "fractal dimension" OR "branching angle" OR parameter* OR architecture* ) ) ) ) OR ( macula* W/2 ( thin* OR thick* ) ) OR "foveal avascular zone" OR "peripapillary retinal nerve fiber*" OR "parafoveal capillary" ) AND ( INDEXTERMS ( "diabetes mellitus" OR "prediabetic state" OR "imparied glucose tolerance" ) OR TITLE-ABS-KEY ( diabet* OR prediabet* OR pre-diabet* OR ( glucose W/1 ( tolerance OR intolerance ) ) ) ) ) OR ( TITLE-ABS-KEY ( retina* W/3 ( marker* OR biomarker OR alteration* OR damage* OR change* OR complicat* OR abnormal* OR function* OR dysfunction* ) ) AND TITLE-ABS-KEY ( ( diabetes OR prediabetes OR prediabetic OR pre-diabetes OR pre-diabetic OR ( glucose W/1 ( tolerance OR intolerance ) ) ) W/5 ( risk OR risks OR incidence* OR predict* OR associat* OR correlate* ) ) ) ) AND NOT ( INDEXTERMS ( ( ( adults OR adult OR adolescent OR child OR juvenile OR infant ) AND NOT ( adult OR adults ) ) OR ( ( human OR humans OR animal OR animals ) AND NOT ( human OR humans ) ) ) )

### Web of Science (4745)

Indexes=SCI-EXPANDED, SSCI, A&HCI, CPCI-S, CPCI-SSH, BKCI-S, BKCI-SSH, ESCI, CCR-EXPANDED, IC Timespan=All years

((ts=((retina* NEAR/2 ((capillar* NEAR/2 (dense or density)) or ((arterio-venous or artery or arterial or arteriolar or arteriole* or vein or venous or venular or venule* or vasculature or vascular or microvascular or macrovascular or vessel*) NEAR/2 (change* or alteration* or ratio* or diameter* or narrow* or dilat* or caliber or calibre or geometry or geometrical or tortuosity or tortuous or "fractal dimension" or "branching angle" or parameter* or architecture*)))) or (macula* NEAR/2 (thin* or thick*)) or "foveal avascular zone" or "peripapillary retinal nerve fiber*" or "parafoveal capillary") AND (ts=(diabet* or prediabet* or pre-diabet* or (glucose NEAR/1 (tolerance or intolerance))))) OR (ts=(retina* NEAR/3 (marker* or biomarker or alteration* or damage* or change* or complicat* or abnormal* or function* or dysfunction*)) AND ts=((diabetes or prediabetes or prediabetic or pre-diabetes or pre-diabetic or (glucose NEAR/1 (tolerance or intolerance))) NEAR/5 (risk or risks or incidence* or predict* or associat* or correlate*))))

### Cochrane (1001)

| **#** | **Search Statement** | **Hits** |
| --- | --- | --- |
| #1 | ((retina* NEAR/2 ((capillar* NEAR/2 (dense or density)) or ((arterio-venous or artery or arterial or arteriolar or arteriole* or vein or venous or venular or venule* or vasculature or vascular or microvascular or macrovascular or vessel*) NEAR/2 (change* or alteration* or ratio* or diameter* or narrow* or dilat* or caliber or calibre or geometry or geometrical or tortuosity or tortuous or fractal dimension or branching angle or parameter* or architecture*)))) or (macula* NEAR/2 (thin* or thick*)) or foveal avascular zone or peripapillary retinal nerve fiber* or parafoveal capillary) | 2143 |
| #2 | MeSH descriptor: [Diabetes Mellitus] explode all trees | 46225 |
| #3 | MeSH descriptor: [Prediabetic State] explode all trees | 1740 |
| #4 | (diabet* or prediabet* or pre-diabet* or (glucose NEAR/1 (tolerance or intolerance))) | 134127 |
| #5 | #2 or #3 or #4 | 134165 |
| #6 | #1 and #5 | 963 |
| #7 | (retina* NEAR/3 (marker* or biomarker or alteration* or damage* or change* or complicat* or abnormal* or function* or dysfunction*)) | 1954 |
| #8 | ((diabetes or prediabetes or prediabetic or pre-diabetes or pre-diabetic or (glucose NEAR/1 (tolerance or intolerance))) NEAR/5 (risk or risks or incidence* or predict* or associat* or correlate*)) | 23566 |
| #9 | #7 and #8 | 74 |
| #10 | #6 or #9 | 1012 |
|  | Limit to Review and Trials | 1001 |

### CINAHL (564)

TI ( ((((retina* N2 ((capillar* N2 (dense or density)) or ((arterio-venous or artery or arterial or arteriolar or arteriole* or vein or venous or venular or venule* or vasculature or vascular or microvascular or macrovascular or vessel*) N2 (change* or alteration* or ratio* or diameter* or narrow* or dilat* or caliber or calibre or geometry or geometrical or tortuosity or tortuous or "fractal dimension" or "branching angle" or parameter* or architecture*)))) or (macula* N2 (thin* or thick*)) or "foveal avascular zone" or "peripapillary retinal nerve fiber*" or "parafoveal capillary") AND ((diabet* or prediabet* or pre-diabet* or (glucose N1 (tolerance or intolerance))))) OR ((retina* N3 (marker* or biomarker or alteration* or damage* or change* or complicat* or abnormal* or function* or dysfunction*)) AND ((diabetes or prediabetes or prediabetic or pre-diabetes or pre-diabetic or (glucose N1 (tolerance or intolerance))) N5 (risk or risks or incidence* or predict* or associat* or correlate*)))) ) OR AB ( ((((retina* N2 ((capillar* N2 (dense or density)) or ((arterio-venous or artery or arterial or arteriolar or arteriole* or vein or venous or venular or venule* or vasculature or vascular or microvascular or macrovascular or vessel*) N2 (change* or alteration* or ratio* or diameter* or narrow* or dilat* or caliber or calibre or geometry or geometrical or tortuosity or tortuous or "fractal dimension" or "branching angle" or parameter* or architecture*)))) or (macula* N2 (thin* or thick*)) or "foveal avascular zone" or "peripapillary retinal nerve fiber*" or "parafoveal capillary") AND ((diabet* or prediabet* or pre-diabet* or (glucose N1 (tolerance or intolerance))))) OR ((retina* N3 (marker* or biomarker or alteration* or damage* or change* or complicat* or abnormal* or function* or dysfunction*)) AND ((diabetes or prediabetes or prediabetic or pre-diabetes or pre-diabetic or (glucose N1 (tolerance or intolerance))) N5 (risk or risks or incidence* or predict* or associat* or correlate*)))) )

### Proquest Dissertations & Theses Global (165)

title(((((retina* NEAR/2 ((capillar* NEAR/2 (dense or density)) or ((arterio-venous or artery or arterial or arteriolar or arteriole* or vein or venous or venular or venule* or vasculature or vascular or microvascular or macrovascular or vessel*) NEAR/2 (change* or alteration* or ratio* or diameter* or narrow* or dilat* or caliber or calibre or geometry or geometrical or tortuosity or tortuous or "fractal dimension" or "branching angle" or parameter* or architecture*)))) or (macula* NEAR/2 (thin* or thick*)) or "foveal avascular zone" or "peripapillary retinal nerve fiber*" or "parafoveal capillary") AND ((diabet* or prediabet* or pre-diabet* or (glucose NEAR/1 (tolerance or intolerance))))) OR ((retina* NEAR/3 (marker* or biomarker or alteration* or damage* or change* or complicat* or abnormal* or function* or dysfunction*)) AND ((diabetes or prediabetes or prediabetic or pre-diabetes or pre-diabetic or (glucose NEAR/1 (tolerance or intolerance))) NEAR/5 (risk or risks or incidence* or predict* or associat* or correlate*))))) OR abstract(((((retina* NEAR/2 ((capillar* NEAR/2 (dense or density)) or ((arterio-venous or artery or arterial or arteriolar or arteriole* or vein or venous or venular or venule* or vasculature or vascular or microvascular or macrovascular or vessel*) NEAR/2 (change* or alteration* or ratio* or diameter* or narrow* or dilat* or caliber or calibre or geometry or geometrical or tortuosity or tortuous or "fractal dimension" or "branching angle" or parameter* or architecture*)))) or (macula* NEAR/2 (thin* or thick*)) or "foveal avascular zone" or "peripapillary retinal nerve fiber*" or "parafoveal capillary") AND ((diabet* or prediabet* or pre-diabet* or (glucose NEAR/1 (tolerance or intolerance))))) OR ((retina* NEAR/3 (marker* or biomarker or alteration* or damage* or change* or complicat* or abnormal* or function* or dysfunction*)) AND ((diabetes or prediabetes or prediabetic or pre-diabetes or pre-diabetic or (glucose NEAR/1 (tolerance or intolerance))) NEAR/5 (risk or risks or incidence* or predict* or associat* or correlate*)))))

Updated search dates: from 17^th^ July 20204 till 28^th^ January 2026

Initial Search: 1689 additional studies

Additional articles included: 2

**Table S1: Table summarizing related systematic and scoping reviews**

| **Author, year** | **Title** | **Main focus** | **Relation to our review** |
| --- | --- | --- | --- |
| Alqahtani et al, 2025 | The efficacy of artificial intelligence in diabetic retinopathy screening: a systematic review and meta-analysis.  <https://doi.org/10.1186/s40942-025-00670-9> | **Diabetic retinopathy** | Our review focuses on diabetes detection (prevalent and incident) and retinal biomarkers, not diabetic retinopathy grading |
| Yang et al., 2025 | Use of artificial intelligence with retinal imaging in screening for diabetes-associated complications: systematic review  DOI: [10.1016/j.eclinm.2025.103089](https://doi.org/10.1016/j.eclinm.2025.103089) | AI applied to retinal images to detect and predict systemic **diabetes‑related complications** (micro- and macrovascular, and other systemic outcomes), not retinal biomarkers of diabetes itself. | Our review instead catalogues and appraises retinal biomarkers of diabetes and future diabetes risk, across AI and non‑AI methods, and evaluates ethnic/geographic heterogeneity and is **not related to diabetes-related complications.** |
| Kodama et al., 2021 | Predictive ability of current machine learning algorithms for type 2 diabetes mellitus: A meta-analysis  DOI: [10.1111/jdi.13736](https://doi.org/10.1111/jdi.13736) | Meta-analysis of ML models using clinical and biochemical risk factors to predict incident T2D in longitudinal cohorts; **no retinal imaging or retinal biomarkers used.** | Complements our work by showing how non‑imaging ML models perform; in contrast, our review maps specific **retinal biomarkers** (vascular and non‑vascular) and their ethnic/geographic variability in relation to prevalent and incident diabetes. |
| Sobhi et al, 2025 | Artificial intelligence for early detection of diabetes mellitus complications via retinal imaging  DOI: [10.1007/s40200-025-01596-7](https://doi.org/10.1007/s40200-025-01596-7) | AI applications using retinal images for DM-related complications, highlighting advancements beyond DR screening- neuropathy, nephropathy, and atherosclerotic cardiovascular disease, as well as to predict the risk of cardiovascular events. | Our review instead catalogues and appraises retinal biomarkers of diabetes and future diabetes risk, across AI and non‑AI methods, and evaluates ethnic/geographic heterogeneity and is **not related to diabetes-related complications.** |
| Li et al., 2025 | Transformative applications of oculomics-based AI approaches in the management of systemic diseases: A systematic review  <https://doi.org/10.1016/j.jare.2024.11.018> | AI algorithms in managing systemic diseases including diabetes. However, the focus is not on specific retinal vascular or non-vascular biomarkers, instead it focuses on use AI models in general. | Our review focuses on specific retinal biomarkers, identification of which can be crucial for development of AI models with hand-crafted expert driven features that are **inherently interpretable** models, which are more trusted in clinical practice |

**Table S2: Overview of Cross-Sectional and Longitudinal Studies Examining Retinal Biomarkers in Diabetes**

| Sl. No | Author (year) | Country/ Ethnicity | Type of fundus camera | Type of retinal biomarker | Total number participants | Diabetes definition | Use of computer-assisted measurements/ AI (ML/DL) tools | Results | Quality Appraisal |  |
| --- | --- | --- | --- | --- | --- | --- | --- | --- | --- | --- |
| Cross-sectional studies | | | | | | | | | | |
| 1. | Aliahmad et al (2014)^55^ | India | Mydriatic | Vascular (CRAE; CRVE; vessel tortuosity, branching; FAZ Size/shape/ ring area; Grayscale Box Counting Fractal Dimension (FD) | 189  (DM=23; NG=166) | Not defined as per ADA/ WHO criteria | Computer-assisted measurement  (Retinal Image and vasculature Assessment Software (RIVAS) v1.0 for FD) | Mean FD for diabetes: significantly higher 2.4403 (p < 0.001, 95%CI:2.4335-2.4471) compared to mean FD for the control group (2.4227, p < 0.001, 95%CI:2.4201-2.4252). | Good |  |
| 2. | Aliahmad et al (2016) ^54^ | India | Mydriatic | Vascular (CRAE; CRVE; Vessel tortuosity; FD, V/B; ABA; TBA | 45  (DM=13; NG=32) | Not defined as per ADA/ WHO criteria | Computer-assisted tool was used (- mlvessel v1.3, RIVAS v1.0. IVAN software (University of Wisconsin, Madison, WI, USA) | Six retinal vascular parameters are associated with diabetes with no observable DR.  1. Narrow CRAE [Coef: −0.088; SE: 0.018; T: −4.71; p<0.001]  2. Mean tortuosity[Coef: 0.219; SE: 0.027; T: 8.13 ; p<0.001]  3. ABA [Coef: 0.239; SE: 0.038; T: 6.24; p <0.001]  4. SD of angle[Coef: 0.179; SE: 0.040; T: 4.45; p<0.001]  5. Lower VB [Coef: −0.269; SE: 0.030; T: −8.88; p <0.001]  6. TBA[Coef: 0.262; SE: 0.022; T: 11.48 ; p <0.001] | Good |  |
| 3. | Cheung et al (2012)^31^ | Singapore | Mydriatic | Vascular (CRAE; CRVE; vessel tortuosity; Retinal vascular branching, fractal dimension) | 2735  (DM=594; NG=2141) | ADA criteria | Computer-assisted measurement (SIVA, V 1.0) | Retinal arteriolar tortuosity, CRAE and CRVE higher in DM  1. Retinal arteriolar tortuosity (×10^4): No DM [2.81 (2.75–2.86); DM [2.70 (2.61–2.79)], p=0.036  2. Retinal arteriolar caliber (CRAE), µm: No DM [134.3 (133.7–134.9 ); DM [136.3 (135.2–137.3 )], p=0.001  3. Retinal venular caliber (CRVE), µm: No DM [204.4 (203.5–205.3); DM [206.5 (205.0–208.0 )], p=0.009 | Very Good |  |
| 4. | daSilva et al (2015)^32^ | Brazil | Mydriatic | Vascular  (Arteriolar diameter; Venular diameter; AVR; TNA, TNV, sum of arteriole and vein diameters in different quatrants | 52 | Not defined as per ADA/ WHO criteria | Computer-assisted measurement | Patients with DM for 5 or more years had larger venule diameters in the upper temporal quadrant  (141.62±44.44 vs 112.58±32.11 µm; p<0.05). | Satisfactory |  |
| 5. | Dong et al (2016)^33^ | China | Mydriatic | Vascular (Arteriolar diameter; Venular diameter; AVR); non-vascular (Oxygen saturation: A_satO2, V_satO2, and AV_difference) | 177  (DM= 99;  NG=78) | Not defined as per ADA/ WHO criteria | Computer-assisted measurement (Oxymap Analyzer software) | DM with PDR group exhibited statistically significant thinner arterioles and wider venules when compared with the normal group. However, both the NPDR and PDR patients had significantly smaller AVR than the controls  There was a significant decrease in arteriolar diameter, an increase in  venular diameter, and a smaller AVR with increasing T2D duration  **Arteriolar diameter (pixels):** Normal: 13.47 ± 1.19; DM no DR: 13.2 ± 1.12; NPDR: 13.03 ± 1.46; PDR: 11.82 ± 1.47** (p<0.001); **Venular diameter (pixels):** Normal: 16.80 ± 1.68; DM no DR: 17.0 ± 1.62; NPDR: 17.28 ± 1.84; PDR: 17.66 ± 1.44* (p<0.05); **AVR:** Normal: 0.81 ± 0.07; DM no DR: 0.78 ± 0.07; NPDR: 0.76 ± 0.07**; PDR: 0.67 ± 0.07**  In a simple linear regression with retinal vessel  diameter in relation to T2D duration, there was a significant decrease in Arteriolar diameter, an increase in  Venular diameter, and a smaller AVR with increasing T2D duration (p < 0.01). [Arteriolar diameter: β (95 % CI): -0.095 (−0.15 to −0.04); p=0.001, Venular diameter: β (95 % CI): 0.101 (0.038 to 0.163); p=0.002, AVR:-0.01 (−0.013 to −0.008); p<0.001]" | Good |  |
| 6. | Islam et al (2009)**^34^** | Singapore (Malay adults) | Non-mydriatic; Desktop based | Vascular (CRAE; CRVE) | 3004  (DM=682;  NG=2322) | Not defined as per ADA/ WHO criteria | Computer-assisted measurement was used | CRAE was higher for DM in multivariable model.  [SE of mean for Model 2 (adjusted for age and gender plus systolic blood pressure, body mass index, total cholesterol, and current smoking) was 139.2 (0.33) for no T2D and 142.2 (0.61) for T2D (P< 0.001);  Model 3 [adjusted for variables in model 2 and venular (in models of arteriolar calibre) and arteriolar calibres (in models of venular calibre)] SE was 139.1 (0.28) for no T2D and 141.3 (0.52) for T2D (P<0.001).]  CRVE): was not significantly associated | Very Good |  |
| 7. | Jeganathan et al (2009)^35^ | Singapore | Mydriatic; Desktop based | Vascular (CRAE; CRVE) | 3748  (2214-Chinese; 807- Malays; 727-Indians) | WHO criteria | Computer-assisted measurement (IVAN; University of Wisconsin, USA) used | Wider CRAE and CRVE were significantly associated with DM  [Participants with T2D had significantly wider retinal arteriolar and venular diameters, compared to those with NFG. The mean CRAE increased from 143.5 mm (micrometers) in those with NFG to 146.0 mm in persons with T2D (P-trend=0.0005) in Model 2 (adjusted for age, gender, ethnicity (except for ethnicity-specific models), systolic blood pressure, body mass index, total cholesterol, triglycerides, and current smoking.), and the mean CRVE increased from 220.5 mm in individuals with NFG to 223.3 mm in persons with T2D (P-trend= 0.007) in Model 2.  In logistic regression analysis adjusting for variables in Model 2, wider CRAE and CRVE were significantly associated with diabetes. The  multivariable odds ratio (OR) (95% CI) of diabetes per 1-SD increase in CRAE was 1.18 (1.06-1.32) and for a 1-SD increase in CRVE was 1.15 (1.03-1.28).] | Very Good |  |
| 8. | Jiang et al (2024)^36^ | China | Desktop based | Vascular  (AVR; CRAE; CRVE; vessel tortuosity; Fractal dimension; vessel curvature, vessel branching coefficient, Number of first branching, vessel branching angle; vessel angle asymmetry, Length-diameter ratio, vessel asymmetry ratio, Junctional exponent deviation, Vessel length density , Vessel area density | 600  (DM=200; PreDM=200; NG=200) | WHO criteria | AI/ ML/DL tools used (U-Net network with Inception-V3 backbone) | The study identified significant differences across the normal, prediabetes, and diabetes groups in ten retinal vascular parameters. These include the following in DM:  Smaller Artery diameter (p=0.008), lower Fractal dimension (p=0.000), reduced Vein curvature (p=0.003), decrease in C-zone artery branching vessel count (p=0.049), higher C-zone vein branching vessel count (p=0.041), Lower Artery branching angle (p=0.005), lower Vein branching angle (p =0.001), Lower Artery angle asymmetry degree (p=0.003), lower Vessel length density(p=0.000), Lower Vessel area density (p=0.000). | Satisfactory |  |
| 9. | Kifley et al (2007)^37^ | Australia | Mydriatic; Desktop based | Vascular (Arteriolar diameter; Venular diameter) | 3,368  (DM=255;  NG=3113) | WHO criteria | Computer-assisted measurement | Arteriolar Caliber was higher for DM compared to Normal. [Adjusted Mean (SE) Standard Error (Adjusted for age, gender, smoking (current, past), systolic blood pressure, hemoglobin, fibrinogen, white cell count, body mass index, alcohol intake, and lipid levels (triglyceride, high-density lipoprotein) of Arteriolar Caliber was higher for DM compared to Normal (DM=193.5 (1.3); N=190.2 (0.4), p=0.01).]  Venular Caliber was not significantly different | Very Good |  |
| 10. | Li et al (2022)^38^ | China | Non-mydriatic; Desktop based` | Vascular  (Monofractal dimension (*Dbox*), multifractal dimension (*D0*), vessel area ratio (*R*), max vessel diameter (*dmax*), average vessel diameter (*dave*), arc–chord ratio (*A/C*), and tortuosity (*τn*)) | 234  (DM=102;  NG=132) | WHO criteria | AI/ ML/DL tools used  (Dense-block generative adversarial network (D-GAN)-assisted retinal vascular segmentation system; analyzed by customized software (ocular microvascular analysis system V2.9.1) for automatic  processing of vessel geometry parameters | DM patients had lower Dbox and D0 values (1.330±0.041; 1.347±0.038) than healthy subjects (1.343±0.048, p<0.05; 1.362±0.042, p<0.05) and showed increasing values of dmax, dave, A/C, and τn compared with normal controls, although only the differences in dave and τn between the groups were statistically significant.  In the regression analysis, dave and τn showed a good correlation with diabetes (dave, OR 1.765, 95% CI 1.319–2.362, p<0.001; τn, OR 9.323, 95% CI 1.492–58.262, p<0.05).  The AUC of the model was 0.713 (95% CI 0.647–  0.780, p<0.001) | Satisfactory |  |
| 11. | Li et al (2020)^39^ | Netherlands | Mydriatic; Desktop based | Vascular (CRAE; CRVE) | 2876  (DM=813;  PreDM= 433;  NG=1630) | WHO criteria | Computer-assisted measurement (RHINO, RetinaCheck group of the Technical University of Eindhoven) was used | CRAE was higher in DM [β =2.89 (0.69, 5.08)]  CRVE was not significantly associated with DM. | Very Good |  |
| 12. | Mozolewska-Piotrowska et al (2019)^40^ | Poland | Desktop based | Vascular (CRAE; CRVE) | 84  (DM=40 (80 eyes);  NG=44 (88 eyes)) | Not defined as per ADA/ WHO criteria | Computer-assisted measurement (VISUALIS and VesselMap Software) | No significant difference between the groups (p=0.85) | Satisfactory |  |
| 13. | Nguyen et al (2008)^41^ | United States | Non-mydriatic; Desktop based | Vascular (CRAE; CRVE) | 5976  (DM with DR= 165; DM without DR= 727; IFG=499; NG=4585) | WHO criteria | Computer-assisted measurement | After multivariate analysis, retinal arteriolar and venular caliber higher among DM (143.8 for NG vs 146.1Microns in diabetes (P= 0.001 for trend))..  In a subgroup analysis by ethnicity, the association between wider arteriolar caliber and DM was evident in whites only, whereas wider venular caliber and diabetes was evident in Hispanics and Chinese only. | Very Good |  |
| 14. | Nikolaidou et al (2022)^67^ | Greece | Non-mydriatic; Desktop based | Vascular (CRAE; CRVE; AVR) | 177  (DM=87; NG=90) | WHO criteria | Computer-assisted tool was used | No significant differences in CRAE, CRVE, and AVR between DM and NG | Good |  |
| 15. | O’Neill et al (2022)^43^ | United Kingdom | Mydriatic; Desktop based | Vascular (CRAE; CRVE; vessel tortuosity; fractal dimension) | 1762  (DM=209;  NG=1553) | Not defined as per ADA/ WHO criteria | Computer-assisted measurement (VAMPIRE, Universities of Dundee and Edinburgh, Scotland, Version 3.1) | AVR, arteriolar tortuosity and venular tortuosity were significantly associated with DM (minimally adjusted odds ratio [OR]=0.85; 95% confidence intervals [CIs] 0.73, 0.99; P=0.04, OR=1.18; 95% CI 1.02, 1.37; P=0.03 and OR=1.20; 95% CI 1.04, 1.38; P=0.01, respectively).  Following adjustment for potential confounders- none were significant. | Very Good |  |
| 16. | Phan et al (2018)^44^ | Australia | Mydriatic; Desktop based | Vascular (CRAE; CRVE) | 748 (IFG=96; ; DM=189; NG=463) | Not defined as per ADA/ WHO criteria | Computer-assisted tool VAN (University of Wisconsin, Madison) was used | In men, wider retinal venular calibre (second and third tertile) significantly higher odds of DM  [multivariable-adjusted OR 2.447, p = 0.005; and OR 2.76, p = 0.002; respectively]  No equivalent association was apparent in women. | Good |  |
| 17. | Sasongko et al (2011)^45^ | Australia | Desktop based | Vascular (Arteriolar diameter; Venular diameter; arteriolar tortuosity; venular tortuosity) | 347  (DM=244; NG=103) | Not defined as per ADA/ WHO criteria | Computer-assisted measurement (SIVA) was used | Mean arteriolar (18.5±8.09 vs 34.4±18.6, p<0.001) & venular tortuosity (22.2±9.15 vs 39.7±18.8, p <0.001) were higher in DM compared with those without.  In the multivariate analysis, retinal arteriolar and venular tortuosity were increased in DM without DR compared with non-diabetic participants. (mean difference 12.4×10−5 and 13.3×10−5, respectively; both p<0.05) | Good |  |
| 18. | Tikellis et al (2007)^46^ | Australia | Non-mydriatic; Desktop based | Vascular (CRAE; CRVE) | 1998  (DM=657;  IGT/IFG=960  NG=381) | WHO criteria | Computer-assisted measurement: Retinal Analysis; Optimate, Madison, WI, USA | After multivariable adjustment, retinal arteriolar calibre was significantly larger in people with known diabetes (178.9 μm) compared with participants with NGT (174.6 μm, p=0.02).  One SD increase in mean arteriolar calibre was associated with higher odds of diabetes compared with NGT (odds ratio [OR]=1.28, 95%CI=1.06–1.55). | Very Good |  |
| 19. | Tsai et al (2011)^47^ | Singapore (Singapore ethnic Indians) | Non-mydriatic; Desktop based | Vascular (CRAE; CRVE) | 3043 | Not defined as per ADA/ WHO criteria | Computer-assisted measurement | Wider CRAE was associated with DM after multivariate adjustment (145.23 mm vs 142.38 mm, P < 0.001) | Very Good |  |
| 20. | Von Hanno et al (2011)^48^ | Norway | Desktop based | Vascular (CRAE; CRVE) | 955  (DM= 317;  NG=638) | Not defined as per ADA/ WHO criteria | Computer-assisted measurement | In multivariable-adjusted analysis, DM was significantly associated with increased CRAE in women (β:2.92 µm, 95% CI:0.71,5.14, p=0.01), but not in men (β: 1.50 µm, 95% CI –0.63,3.63, p=0.08).  . | Unsatisfactory |  |
| 21. | Yau et al (2010)^49^ | Australia | Non-mydriatic; Desktop based | Vascular (fractal dimension (Df)) | 1577 | WHO criteria | Computer-assisted measurement | After adjustment, persons with higher retinal Df were more likely to have DM.[OR 1.56; 95% CI 1.14–2.14, highest vs lowest fractal tertile] | Very Good |  |
| 22. | Zee et al. (2022)^50^ | China | Non-mydriatic; Desktop based | Vascular  (CRAE, CRVE, vessel tortuosity, Texture-related features, fractal-related features such as fractal dimension, and spectrum-related feature) | 2221  (DM=945; NG=1276) | Not defined as per ADA/ WHO criteria | AI/ ML/DL tools used  (Automatic retinal image analysis (ARIA) program; ‘inceptionResNet’-convolutional neural network from Matlab | In DM, retinal arteries and veins tend to be slightly wider (CRAE, CRVE). There is an increase in tortuosity. There is a higher prevalence of hemorrhages, occlusions, and exudates. The arteriolar-venous nipping feature is less pronounced in DM.  Mean bifurcation coefficient of arterioles (MBCA) is higher, the bifurcation angles of arterioles (MAangle) and venules (MVangle) are increased  The 10-fold cross-validation using the support vector machine approach has a sensitivity of 92% and a specificity of 96.2%. The separate testing data from Hong Kong provided a sensitivity of 99.5% (95% CI: 99.0 to 100.0) and a specificity of 91.1%. (95%CI: 89.1 to 93.2) For the UK testing data, the sensitivity is 98.0%. | Satisfactory |  |
| 23. | Zhang et al. (2018)^51^ | Netherlands | Non-mydriatic; Desktop based | Vascular (CRAE; CRVE; fractal dimension) | 1943 | Not defined as per ADA/ WHO criteria | Computer-assisted measurement  (RHINO) | None of the artery (AV) biomarkers (CRAE (AVR), Artery (AV) tortuosity, box dimension, lacunarity, Box/lacunarity ratio) were significantly different.  The following were significantly different CRVE (wider) (p=0.027), vein tortuosity (increased) (p=0.04) | Satisfactory |  |
| 24. | Zhang et al (2023)^52^ | China | Non-mydriatic | Vascular (optical density ratio (ODR), ODR variability (ODRv)) | 100  (DM=50; NG=50) | Not defined as per ADA/ WHO criteria | AI/ ML/DL tools used  (U-net by employing a multipath attention network model (MA-net)) | The ODRs of all vascular subgroups except the micro venule were significantly higher; ODRv was significantly lower in NDR group than that in healthy normal group.  In the regression analysis, the increased ODRs except micro venule and decreased ODRv were significantly correlated with the incidence of DM.  C-statistic for discrimination DM with all ODR is 0.777 (95% CI 0.687-0.867, p < 0.001). | Unsatisfactory |  |
| 25. | Zhang et al (2024)^53^ | China | Non-mydriatic; Desktop based | Vascular (Df, vessel area ratio (VAR), mean vessel diameter (D), tortuosity (τ), ODR | 277  (DM=166  NG=111) | ADA criteria | AI/ ML/DL tools used (  Pytorch, Python 3.6 (Python Software Foundation, Hampton, NH), Matlab 2022a (MathWorks Inc., Natick, MA, USA). | Increased main vessels ODR [OR, 1.42; 95%CI, [1.07–1.89]; p = 0.016], decreased VAR of all areas except the outer ring macula [0.57; [0.40–0.81]; p < 0.001)], smaller all vessels mean vascular diameter-Dm (0.66; [0.49–0.89]; p = 0.006), and decreased vessel tortuosity-τ of all areas (e.g. full macula: 0.53; [0.38–0.74]; p < 0.001) were associated NDR (diabetes with no clinically detectable retinopathy (NDR)). | Satisfactory |  |
| 26. | Chen et al  (2025)^57^ | China | Desktop based | CRAE; CRVE; arteriolar tortuosity; venular tortuosity.  Fractal dimensions; branching angle | 320  (NG= 54  PreDM= 71)  T2DM without DR = 144  T2DM+mild/ moderate NPDR changes = 51). | Chinese guidelines | AI/ ML/DL tools used  HR-Net (High-Resolution Network) | Venous fractal dimensions, venous tortuosity, vessel density were lower among DM without DR vs no DM whereas arterial length was higher  In the T2DM without DR group, the average venous tortuosity was lower than in the non-diabetic group. | Good |  |
| 27. | Kaup et al  (2026)^56^ | India | Mydriatic; Desktop based | Arteriolar tortuosity; venular tortuosity; vessle tortuosity; curvature, angle, tortuosity, branch statistics, and pixel connectivity from arteries and veins | 139  (NG=19,  PreDM= 20, DM= 100) | WHO criteria | AI/ ML/DL tools used  Unet, nnUnet, Computer vision tools | DM was associated with greater retinal venous curvature, thicker vessel calibers, and greater variability in vessel thickness. Branching patterns were also more irregular, as reflected by the lower kurtosis of branch angle variability. In this context, “maximum branch thick-  ness” denotes the largest vessel caliber observed along any single venous branch in the segmented vascular tree, and “kurtosis of branch angle variability” denotes the kurtosis of the distribution of bifurcation angles across all venous branch points in an eye, which quantifies how concentrated or dispersed the branching angles are. In contrast, the normoglycemia group demonstrated relatively smoother and thinner vessels, with branch angles that were more uniform and consistent across the retinal images. | Very good |  |
| Longitudinal studies | | | | | | | | | | |
| 1. | Ikram et al (2006)^58^ | Netherlands | Desktop based | Vascular (CRAE; CRVE) | 2309 | WHO criteria | Computer-assisted measurement (Retinal Analysis; Optimate, Madison, WI) | Smaller Arteriolar diameter (OR 1.08 (0.89–1.31) or larger venular diameter (OR 1.09 (0.90–1.33) were not associated with DM | Good |  |
| 2. | Kifley et al (2008)^59^ | Australia | Mydriatic | Vascular (CRAE; CRVE) | 3654 | WHO criteria | Computer-assisted measurement (Retinal Analysis, Optimate, Madison, Wisconsin, USA) | The association of arteriolar and venular caliber with incident diabetes- non significant.  [Adj OR: 1.12 (0.89–1.41)].  In participants younger than 70 years, the association between wider retinal venular caliber and combined endpoint (IFG + DM) was significant. | Good |  |
| 3. | Nguyen et al (2008)^60^ | Australia | Non-mydriatic | Vascular (CRAE; CRVE) | 803  (IFG=66; IGT=491; NG=246) | WHO criteria | Computer-assisted measurement | Narrower retinal arteriolar caliber predicted risk of DM.  [Multivariate analysis, participants with narrower retinal arteriolar caliber had a higher risk of incident diabetes (odds ratio 2.21 [95% CI 1.02–4.80], comparing smallest versus highest arteriolar caliber tertiles, P =0.04 for trend)] | Good |  |
| 4. | Tan et al (2019)^61^ | Singapore (Chinese, Malays and Indians ) |  | Vascular (CRAE; CRVE; vessel tortuosity, branching; fractal dimension) | 4992 | ADA criteria | Computer-assisted measurement (SIVA) used | After multivariate regression, those with greater arteriole calibre [HR: 1.03; p<0.001] and greater venous calibre [HR: 1.01; p=0.013] were more likely to develop DM. | Good |  |
| 5. | Wong et al (2002)^63^ | United States | Non-mydriatic | Vascular  (Arteriolar diameter; Venular diameter; AVR) | 7993 | ADA criteria | Computer-assisted measurement (Khoros, University of New Mexico - Albuquerque). | The incidence of diabetes was higher in persons with lower AVR at baseline. [OR: 1.71; 95% CI: 1.13-2.57; P for trend=.002]  Retinal arteriolar narrowing is independently associated with risk of DM. | Good |  |
| 6. | Wong et al (2005)^62^ | United States | Desktop based | Vascular (CRAE; CRVE) | 3251 | ADA criteria | Computer-assisted measurement (program based on microdensitometric methods) | Participants with narrower retinal arteriolar diameters had a higher incidence of DM.  Multivariable-adjusted model: Smaller AVR higher risk of DM [Multivariable-Adj RR=1.53; 95% CI: 1.03-2.27; comparing smallest to largest arteriole-to-venule ratio quartiles);  Participants with both hypertension and retinal arteriolar narrowing had a 3-fold higher risk of incident DM than normotensive participants without arteriolar narrowing. [Multivariable-Adj RR=3.41; 95% CI, 1.66-6.98]. | Good |  |
| 7. | Yau et al (2012)^64^ | Australia | Non-mydriatic; Desktop based | Vascular (CRAE; CRVE) | 6814 | WHO criteria | Computer-assisted measurement (IVAN; University of Wisconsin, USA) used | Participants with wider retinal arteriolar caliber had a higher risk of developing DM. [HR: 1.60; 95% CI: 1.12–2.29, p = 0.011 comparing highest with lowest arteriolar calibre tertile].  In ethnic subgroup analysis, the association between wider retinal arteriolar caliber and incident DM was stronger and statistically significant only in Caucasians [HR: 2.78; 95% CI: 1.37–5.62, p = 0.005].  Retinal venular calibre and retinopathy signs were not related to risk of diabetes or IFG. | Good |  |

CRAE= Central retinal artery equivalent; CRVE= Central retinal vein equivalent; AVR= Arterio-venous ratio; IFG= Impaired Fasting Glucose;IGT= Impaired Glucose Tolerence; DM= Diabetes Mellitus; PreDM= Prediabetes; NG=Normoglycemia; FD= Fractal Dimensions; V/B= vessel-to-background ratio; ABA= acute branching angles; TBA= total number of branching angles; DR=Diabetic Retinopathy; TNA=total number of arterioles; TNV= total number of venules; Oxygen saturation of the retinal arteriole, veins and their difference (A_satO2, V_satO2, and AV_difference); FAZ= Foveal Avascular Zone

**Table S3: Critical Appraisal of included Cross-sectional Studies Using the Modified Newcastle–Ottawa Scale**

| **Sl No** | | **Study (Author, Year)** | | **Selection** | | | | | | | | | **Comparability:**  **Confounder Control** | | **Outcome** | | | | **Overall**  **Score** | | **Judgment** | |  |
| --- | --- | --- | --- | --- | --- | --- | --- | --- | --- | --- | --- | --- | --- | --- | --- | --- | --- | --- | --- | --- | --- | --- | --- |
|  |  |  |  | **Represent-**  **ativeness** | | **Sample Size** | | **Non-respondents** | | **Exposure**  **Ascertainment** | | |  |  | **Assessment** | | **Statistical Test** | |  |  |  |  |  |
| 1 | | Aliahmad B et al (2014)^58^ | |  | |  | |  | |  | | |  | |  | |  | |  | | Good | |  |
| 2 | | Aliahmad B et al (2016) ^57^ | |  | |  | |  | |  | | |  | |  | |  | |  | | Good | |  |
| 3 | | Cheung CY et al (2012)^42^ | |  | |  | |  | |  | | |  | |  | |  | |  | | Very Good | |  |
| 4 | | daSilva AVB et al (2015)^56^ | |  | |  | |  | |  | | |  | |  | |  | |  | | Satisfactory | |  |
| 5 | | Dong Y et al (2016)^50^ | |  | |  | |  | |  | | |  | |  | |  | |  | | Good | |  |
| 6 | | Islam FMA et al (2009)^71^ | |  | |  | |  | |  | | |  | |  | |  | |  | | Very Good | |  |
| 7 | | Jeganathan VSE et al (2009)^43^ | |  | |  | |  | |  | | |  | |  | |  | |  | | Very Good | |  |
| 8 | | Jiang Y et al (2024)^51^ | |  | |  | |  | |  | | |  | |  | |  | |  | | Satisfactory | |  |
| 9 | | Kifley A et al (2007)^30^ | |  | |  | |  | |  | | |  | |  | |  | |  | | Very Good | |  |
| 10 | | Li M et al (2022)^52^ | |  | |  | |  | |  | | |  | |  | |  | |  | | Satisfactory | |  |
| 11 | | Li W et al (2020)^59^ | |  | |  | |  | |  | | |  | |  | |  | |  | | Very Good | |  |
| 12 | | Mozolewska-Piotrowska K et al (2019)^40^ | |  | |  | |  | |  | | |  | |  | |  | |  | | Satisfactory | |  |
| 13 | | Nguyen TT et al (2008)^47^ | |  | |  | |  | |  | | |  | |  | |  | |  | | Very Good | |  |
| 14 | | Nikolaidou B et al (2022)^34^ | |  | |  | |  | |  | | |  | |  | |  | |  | | Good | |  |
| 15 | | O’Neill R A et al (2022)^46^ | |  | |  | |  | |  | | |  | |  | |  | |  | | Very Good | |  |
| 16 | | Phan K et al (2018)^26^ | |  | |  | |  | |  | | |  | |  | |  | |  | | Good | |  |
| 17 | | Sasongko MB et al (2011)^27^ | |  | |  | |  | |  | | |  | |  | |  | |  | | Good | |  |
| 18 | | Tikellis G et al (2007)^28^ | |  | |  | |  | |  | | |  | |  | |  | |  | | Very Good | |  |
| 19 | | Tsai ASH et al (2011)^44^ | |  | |  | |  | |  | | |  | |  | |  | |  | | Very Good | |  |
| 20 | | Von Hanno T et al (2011)^39^ | |  | |  | |  | |  | | |  | |  | |  | |  | | Unsatisfactory | |  |
| 21 | | Yau JWY et al (2010)^32^ | |  | |  | |  | |  | | |  | |  | |  | |  | | Very Good | |  |
| 22 | | Zee B et al. (2022)^53^ | |  | |  | |  | |  | | |  | |  | |  | |  | | Satisfactory | |  |
| 23 | | Zhang J et al. (2018)^38^ | |  | |  | |  | |  | | |  | |  | |  | |  | | Satisfactory | |  |
| 24 | | Zhang J et al (2023)^54^ | |  | |  | |  | |  | | |  | |  | |  | |  | | Unsatisfactory | |  |
| 25 | | Zhang J et al (2024)^55^ | |  | |  | |  | |  | | |  | |  | |  | |  | | Satisfactory | |  |
| 26 | | Chen N et al  (2025)^56^ | |  | |  | |  | |  | | |  | |  | |  | |  | | Good | |  |
| 27 | | Kaup S et al  (2026)^65^ | |  | |  | |  | |  | | |  | |  | |  | |  | | Very Good | |  |
| **Longitudinal Studies: Critical Appraisal of included Longitudinal Studies Using the Newcastle–Ottawa Scale for Cohort studies.** | | | | | | | | | | | | | | | | | | | | | | | |
| **Sl No** | **Study (Author, Year)** | | **Selection** | | | | | | | | **Comparability** | | | **Outcome** | | | | | | **Overall Score (max 9)** | | **Quality Judgement** | |
|  |  |  | **Exposed Cohort** | | **Non-exposed Cohort** | | **Ascertainment of Exposure** | | **Outcome Not Present at Start** | | **Controlled Confounders** | **Other Factors** | | **Assessment** | | **Follow-up Duration long enough** | | **Adequacy of Follow-up** | |  |  |  |  |
| 1 | Ikram MK et al (2006)^37^ | |  | |  | |  | |  | |  |  | |  | |  | |  | |  | | Good | |
| 2 | Kifley A et al (2008)^31^ | |  | |  | |  | |  | |  |  | |  | |  | |  | |  | | Good | |
| 3 | Nguyen TT et al (2008)^29^ | |  | |  | |  | |  | |  |  | |  | |  | |  | |  | | Good | |
| 4 | Tan GS et al (2019)^45^ | |  | |  | |  | |  | |  |  | |  | |  | |  | |  | | Good | |
| 5 | Wong TY et al (2002)^48^ | |  | |  | |  | |  | |  |  | |  | |  | |  | |  | | Good | |
| 6 | Wong TY et al (2005)^49^ | |  | |  | |  | |  | |  |  | |  | |  | |  | |  | | Good | |
| 7 | Yau JWY et al (2012)^60^ | |  | |  | |  | |  | |  |  | |  | |  | |  | |  | | Good | |

**Figure S1. Example of a retinal colour photograph and the use of artificial intelligence (AI)/machine learning (ML) tools for vessel segmentation, artery–vein classification, and feature extraction.**
